# HEMIGEN: Human Embryo Image Generator Based on Generative Adversarial Networks

**DOI:** 10.3390/s19163578

**Published:** 2019-08-16

**Authors:** Darius Dirvanauskas, Rytis Maskeliūnas, Vidas Raudonis, Robertas Damaševičius, Rafal Scherer

**Affiliations:** 1 Department of Multimedia Engineering, Kaunas University of Technology, 51368 Kaunas, Lithuania; 2 Department of Control Systems, Kaunas University of Technology, 51367 Kaunas, Lithuania; 3 Department of Software Engineering, Kaunas University of Technology, 51368 Kaunas, Lithuania; 4 Institute of Mathematics, Silesian University of Technology, 44-100 Gliwice, Poland; 5 Institute of Computational Intelligence, Czestochowa University of Technology, 42-200 Czestochowa, Poland

**Keywords:** deep learning, neural network, generative adversarial network, synthetic images

## Abstract

We propose a method for generating the synthetic images of human embryo cells that could later be used for classification, analysis, and training, thus resulting in the creation of new synthetic image datasets for research areas lacking real-world data. Our focus was not only to generate the generic image of a cell such, but to make sure that it has all necessary attributes of a real cell image to provide a fully realistic synthetic version. We use human embryo images obtained during cell development processes for training a deep neural network (DNN). The proposed algorithm used generative adversarial network (GAN) to generate one-, two-, and four-cell stage images. We achieved a misclassification rate of 12.3% for the generated images, while the expert evaluation showed the true recognition rate (TRR) of 80.00% (for four-cell images), 86.8% (for two-cell images), and 96.2% (for one-cell images). Texture-based comparison using the Haralick features showed that there is no statistically (using the Student’s t-test) significant (*p* < 0.01) differences between the real and synthetic embryo images except for the sum of variance (for one-cell and four-cell images), and variance and sum of average (for two-cell images) features. The obtained synthetic images can be later adapted to facilitate the development, training, and evaluation of new algorithms for embryo image processing tasks.

## 1. Introduction

Deep neural networks (DNN) have become one of the most popular modern tools for image analysis and classification [1]. One of the first accurate implementations of DNN, AlexNet [2], was quickly bested by a deep convolutional activation feature (DeCAF) network, which extracted features from AlexNet and evaluated the efficacy of these features on generic vision tasks [3]. VGGNet demonstrated that increasing the depth of convolutional neural network (CNN) is beneficial for the classification accuracy [4]. However, the deeper neural network is more difficult to train. ResNet presented a residual learning framework to ease the training for very deep networks [5]. The ResNet architecture, which combined semantic information from a deep, coarse layer with appearance information from a shallow network, was adapted for image segmentation [6]. Region CNN (R-CNN) was proposed as a combination of high-capacity CNNs with bottom-up region proposals in order to localize and segment images [7].

The breakthrough of DNNs in quality led to numerous adaptations in solving medical image processing problems such as the analysis of cancer cells [8] and cancer type analysis [9]. Deep max-pooling CNNs were used to detect mitosis in breast histology images using a patch centered on the pixel as context [10]. The U-Net architecture with data augmentation and elastic deformations achieved very good performance on different biomedical segmentation applications [11]. A supervised max-pooling CNN was trained to detect cell pixels in regions that are preselected by a support vector machine (SVM) classifier [12]. After a pre-processing step to remove artefacts from the input images, fully CNNs were used to produce the embryo inner cell mass segmentation [13]. A set of Levenberg–Marquardt NNs trained using textural descriptors allowed for predicting the quality of embryos [14]. 

Image analysis methods were also applied to embryo image analysis. Techniques for extracting, classifying, and grouping properties were used to measure the quality of embryos. Real-time grading techniques for determining the number of embryonic cells from time-lapse microscope images help embryologists to monitor the dividing cells [15]. Conditional random field (CRF) models [16] were used for cell counting and assessing various aspects of the developing embryo and predicting the stage of the embryonic development. In addition to grading, embryonic positioning was achieved using linear chain Markov model [17]. Different solutions were developed for segmenting and calculating the number of cells: ImageJ, MIPAV, VisSeg [18]. Moreover, cell segmentation by marking its center and edges makes it possible to determine their shapes and quantities more quickly and accurately [19]. Two-stage classifier for embryo image classification was proposed in [20].

The rising progress of generative adversarial networks (GANs) applications on medical imaging state that most of the research is focused on synthetic imaging, the reconstruction, segmentation, and classification and proves the importance of the sector. Wasserstein-based GANs were applied for the synthesis of cells imaged by fluorescence microscopy capturing these relationships to be relevant for biological application [21]. The quality of generated artificial images also improved due to the improvements to deep neural networks [22] as well as on progressive growing of GANs for image data augmentation [23]. Variational autoencoders (VAE) and generative adversarial networks (GANs) are currently the most distinguished according to the quality of their results. The VAE models are more often used for image compression and recovery. Both methods have drawbacks, as recovering a part of the information loses some data and often introduces image fading or blurring. This effect could be reduced by matching the data as well as the loss distributions of the real and fake images by a pair of autoencoders used as the generator and the discriminator in the adversarial training [24]. Utilization of both generator and discriminator growing progressively from a low-resolution standpoint and adding new layers for an increase in fine details allowed for achieving a current state-of-the-art inception score of 8.80 on CIFAR10 dataset [25]. Network modifications can be applied to minimize fading of the resulting images [24]. Generative stochastic networks (GSN) were used to learn the transition operator of a Markov chain whose stationary distribution estimates the data distribution [26]. Deep recurrent attentive writer (DRAW) neural network architecture [27] was used to generate highly realistic natural images such as photographs of house numbers or other digits in the MNIST database. Dosovitskiy et al. [28] introduced an algorithm to find relevant information from the existing 3D chair models and to generate new chair images using that information. Denton et al. [29] introduced a generative parametric model capable of producing high-quality samples of natural images based on a cascade of convolutional networks within a Laplacian pyramid framework. Radford and Metz [30] introduced a simplification of training by a modification called deep convolutional GANs (DCGANs). GANs can also recover images from bridging text and image modelling, thus translating visual concepts from characters to pixels [31]. Zhang et al. [32] proposed stacked GANs (StackGAN) with conditioning augmentation for synthesizing photo-realistic images. GANs could also be applied for generating motion images, e.g., the motion and content decomposed GAN (MoCoGAN) framework for video generation by mapping a sequence of random vectors to a sequence of video frames [33]. The GAN for video (VGAN) model was based on a GAN with a spatial-temporal convolutional architecture that untangles the scene’s foreground from the background and can be used at predicting plausible futures of static images [34]. Temporal GAN (TGAN) was used to learn a semantic representation of unlabeled videos by using different types of generators via Wasserstein GAN, and a method to train it stably in an end-to-end manner [35]. MIT presented a 3D generative adversarial network (3D-GAN) model to recreate 3D objects from a probabilistic space by leveraging recent advances in volumetric CNNs and GANs [36]. Li et al. [37] used multiscale GAN (DR-Net) to remove rain streaks from a single image. Zhu [38] used GANs for generating synthetic saliency maps for given natural images. Ma et al. [39] used background augmentation GANs (BAGANs) for synthesizing background images for augmented reality (AR) applications. Han et al. presented a system based on a Wasserstein GANs for generating realistic synthetic brain MR images [40].

All these results are achieved by using large expert-annotated and ready-made databases and also exposing the problem of lacking good, core training datasets. In this work, we aimed to develop a method to generate realistic synthetic images that could later be used for classification, analysis, and training, thus resulting in the creation of novel synthetic datasets for research areas lacking data such as human embryo images. Here we used human embryo images obtained during cell development processes for training a DNN. We propose an algorithm for generating one-, two-, and four-cell images (the selection was based on the initial dataset provided by our medical partners) to increase the overall number of unique images available for training. For generating images, we have developed a generative adversarial network for image recovery, filling, and improvement. The significance of our approach would be that the method was applied to embryonic cell images as a type of image. It was very important for the GAN to accurately reproduce the outline of the cell (often poorly visible even in microscopy picture), since the whole image was almost the same (gray) and the cell itself is translucent. Our focus was not only to generate the generic image of a cell as such, but to make sure that it has all necessary attributes of a real cell image to provide a fully realistic synthetic version. We believe that as a large number of real embryo images required for training neural networks are difficult to obtain due to ethics requirements, the synthetic images generated by the GAN can be later adapted to facilitate the development, training, and evaluation of new algorithms for embryo image-processing tasks.

## 2. Materials and Methods 

### 2.1. Architecture of the Generative Adversarial Network (GAN)

**Figure 1:**
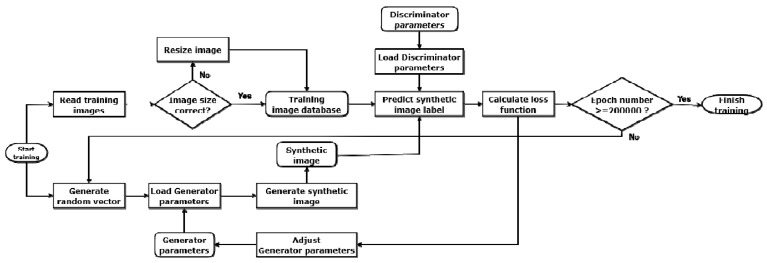
A pipeline of a generative adversarial network (GAN).

The generative adversarial network (GAN) consists of two parts: A generator *G* and a discriminator *D* (Figure 1). 

The discriminator tries to distinguish true images from synthetic, generator-generated images. The generator tries to create images with which the discriminator could be deceived. The discriminator is implemented as a fully connected neural network with dense layers that represents an image as a probability vector and classifies it into two classes, a real or a fake image. The generator is a reverse model that restores the former image from a random noise vector. During the training, the discriminator is trained to maximize the probability of assigning the correct class to the training images and images generated by the generator. At the same time, the generator is trained to minimize classification error log(1−D(G(z))). This process can be expressed in terms of game theory as a two-player (discriminator and generator) minimax game with cost function V(G,D) [41]:

(1) $$\underset{G}{\max}\underset{D}{\min}\;V\left(D,G\right)=E_{x\sim P\left(x\right)}\left[\log\left(D\left(x\right)\right)\right]+E_{z\sim P\left(z\right)}\left[\log\left(1-D\left(G\left(z\right)\right)\right)\right].$$

**Figure 2:**
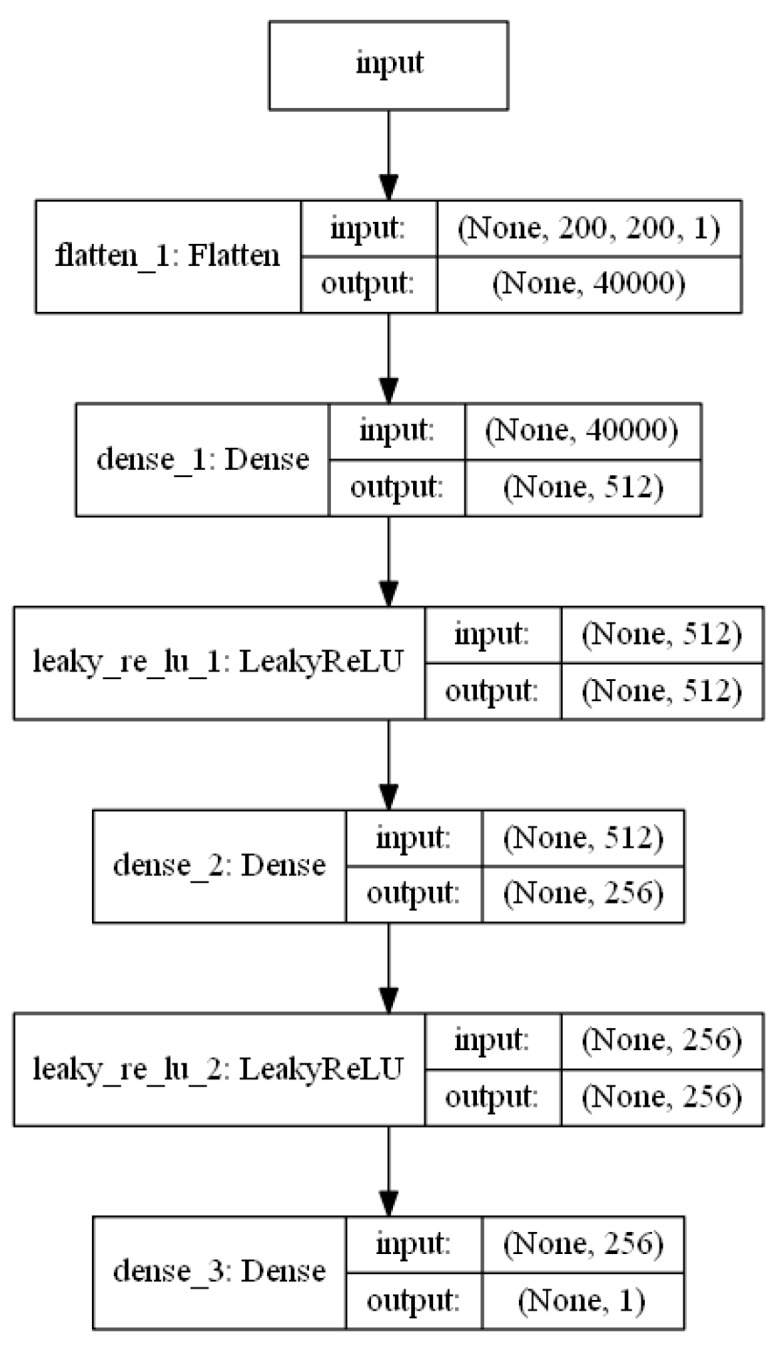
Architecture of the discriminator network.

The discriminator evaluates the images created by the training database and the generator. The architecture of the discriminator network is shown in Figure 2. The network consists of six layers. The monochrome 200 × 200 pixel image in the first layer is transformed and expanded into a single line vector. The dense layer is used in the second, fourth, and sixth layers. The LeakyReLU [42] function, where α = 0.2, is used in the third and fifth layers as it allows a small gradient when the unit is not active:

(2) LReLu(x)=max(x,0)+αmin(x,0).

At the network output, we get a one if the network guesses that the image is real or zero if the network guesses that the image is fake.

**Figure 3:**
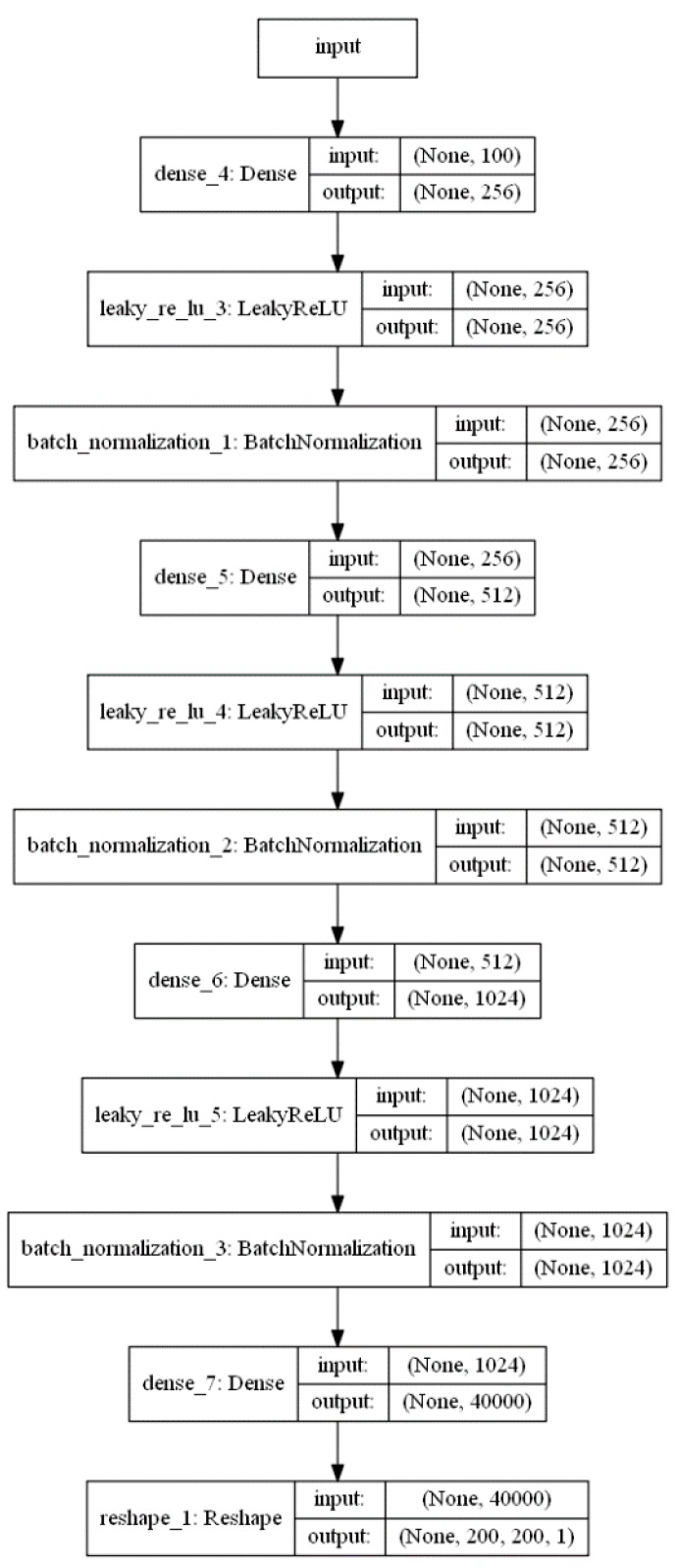
Architecture of the generator network.

The generator is composed of 11 layers (Figure 3). The input layer contains 1 × 100 vector of randomly generated data. These data are transformed using the dense, LeakyReLU, and batch normalization layers. The ReLU layer uses α = 0.2. The batch normalization layer was used to normalize the activations of the previous layer at each batch, by applying a transformation that maintains the mean activation close to 0 and the activation standard deviation close to 1 [43]:

(3) $${\hat{x}}_{i}\leftarrow \frac{x_{i}-\mu_{\beta }}{\sqrt{\sigma_{\beta }^{2}+\varepsilon }}.$$

The batch normalization momentum value was set to 0.8, while all other values were used as per default Keras model values. The output produces a 200 × 200 pixel sized black-and-white image.

### 2.2. Training

We trained one (single) network to generate all types of cell images. For the training, we have used the Adam optimization algorithm [44] with a fixed training speed of 0.00002, and $\beta_{1}$ = 0.7 for training the generator. During training, the Adam algorithm calculates the gradient as follows:

(4) $$g_{t}\leftarrow \Delta_{\theta }f_{t}\left(\theta_{t-1}\right).$$

The biased first moment estimate is updated as follows:

(5) $$m_{t}\leftarrow \beta_{1}m_{t-1}+\left(1-\beta_{1}\right)g_{t}.$$

The biased second moment estimate is update as follows:

(6) $$v_{t}\leftarrow \beta_{2}v_{t-1}+\left(1-\beta_{2}\right)g_{t}^{2}.$$

Then we compute the bias corrected first moment estimate as follows:

(7) $${\hat{m}}_{t}\leftarrow \frac{m_{t}}{1-\beta_{1}^{t}}.$$

The bias corrected second moment estimate is computed as follows:

(8) $${\hat{v}}_{t}\leftarrow \frac{v_{t}}{1-\beta_{2}^{t}}.$$

This gives the update parameters as follows:

(9) $$\theta_{t}\leftarrow \frac{\theta_{t-1}-a{\hat{m}}_{t}}{\sqrt{{\hat{v}}_{t}}\prime \prime +\varepsilon }.$$

The binary cross-entropy function is used to evaluate the error as follows:

(10) $$-\frac{1}{N}\sum_{i=1}^{N}\left[y_{i}\log\left({\hat{y}}_{i}\right)+\left(1-y_{i}\right)\log\left(1-{\hat{y}}_{i}\right)\right].$$

### 2.3. Evaluation of Image Quality 

The problem with using the GAN model is that there are no clear ways to measure it qualitatively. There are few most commonly used criteria to evaluate GAN network: Average log-likelihood, classifier classification [41], and visual fidelity of samples [45]. These methods have both advantages and disadvantages. Log-likelihood and classifiers attribute the image to a certain class with a matching factor. However, they only indicate whether the generated image is like the average of one of the classes but does not value a qualitatively generated image. Such a qualitative assessment requires a human evaluator, but with a large amount of data, such estimates can change independently and be biased [46]. Likewise, a different expert person could evaluate the same data somewhat differently.

We use a combined method to evaluate the results obtained in our study, which includes: (1) Human expert evaluation, (2) histogram comparison, and (3) texture feature comparison. The generated images are evaluated by human experts (three medical research professionals) to determine if they are being reproduced qualitatively. If we can determine its class from the received image, the image is quality-restored. The expert based scoring was calculated using a visual Turing test [47] as this method has already been proven effective in the evaluation of synthetic images generated by GANs [48].

The histogram comparison method checks whether the histogram of the generated images corresponds to the histogram of the images in the training database. For comparison, we used the normalized average histogram of training data $H_{1}$, and the normalized average histogram of generated images $H_{2}$. Further, we apply four different methods for comparison as it is recommended in [49]. The correlation function determines the likelihood of two image histograms. Its value of two identical histograms equals one:

(11) $$C\left(H_{1},H_{2}\right)=\frac{\sum_{I}\left(H_{1}\left(I\right)-{\bar{H}}_{1}\right)\left(H_{2}\left(I\right)-{\bar{H}}_{2}\right)}{\sqrt{{\sum_{I}\left(H_{1}\left(I\right)-{\bar{H}}_{1}\right)}^{2}\sum_{I}{\left(H_{2}\left(I\right)-{\bar{H}}_{2}\right)}^{2}}}.$$

The Chi-square function takes the squared difference between two histograms. The squared differences are divided by the number of samples, and the sum of these weighted squared differences is the likelihood value:

(12) $$\chi^{2}\left(H_{1},H_{2}\right)=\sum_{I}\frac{{\left(H_{1}\left(I\right)-H_{2}\left(I\right)\right)}^{2}}{H_{1}\left(I\right)}.$$

The histogram intersection calculates the similarity of two discretized histograms, with a possible value of the intersection lying between no overlap and identical distributions. It works well on categorical data and deals with null values by making them part of the distribution.

(13) $$I\left(H_{1},H_{2}\right)=\sum_{I}\min\left(H_{1}\left(I\right),H_{2}\left(I\right)\right)$$

The Bhattacharyya distance approximates the normalized distance between the histograms using the maximum likelihood of two object histograms as follows:

(14) $$B\left(H_{1},H_{2}\right)=\sqrt{1-\frac{1}{\sqrt{H_{1}H_{2}N^{2}}}\sum_{I}\sqrt{H_{1}\left(I\right)H_{2}\left(I\right)}}.$$

The texture-based comparison uses the texture analysis features based on grey level co-occurrence matrix (GLCM), which are related to second-order image statistics that were introduced by Haralick [50] as follows: (1) Angular second moment (energy), (2) contrast, (3) correlation, (4) variance, (5) inverse difference moment (homogeneity), (6) sum average, (7) sum variance, (8) sum entropy, (9) entropy, (10) difference variance, (11) difference entropy, (12) information measure of correlation, (13) information measure of correlation II, and (14) maximal correlation coefficient.

### 2.4. Ethics Declaration

The study was conducted in accordance with the Declaration of Helsinki, and the protocol was approved by the Ethics Committee of Faculty of Informatics, Kaunas University of Technology (No. IFEP201809-1, 2018-09-07).

## 3. Experiment and Results

### 3.1. Dataset and Equipment

**Figure 4:**
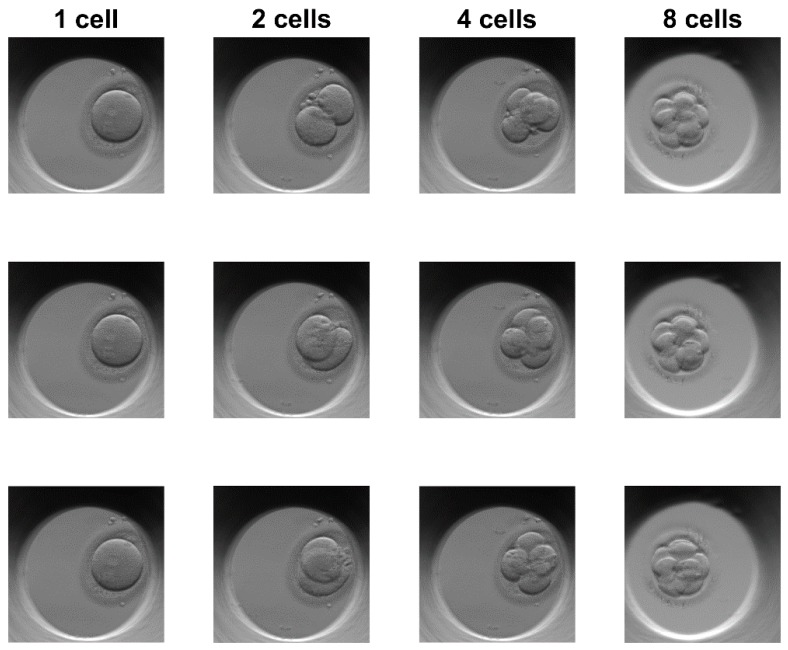
Sample images from Esco embryo image dataset.

We have used the embryo photos taken with Esco Global incubator series, called Miri TL (time lapse). The embryo image sets were registered in the German, Chinese, and Singapore clinics. No identity data were ever provided to the authors of this paper. The Esco’s embryo database consists of three image classes that together have 5000 images with a resolution of 600 × 600 pixels. Number of different images in classes: One-cell—1764 images, two-cell—1938 images, four-cell—1298 images. Images are obtained from 22 different growing embryos in up to five stages of cell evolution. The database was then magnified by rotating each photo at 90, 180, and 270 degrees. Resulting number of different images in classes: (one-cell images—1764 × 4 = 7056; two-cell images—1938 × 4 = 7752; four-cell images—1298 × 4 = 5192). The images were taken in the following culturing conditions: Temperature of 37 °C, stable level of 5% CO2, and the controllable values of nitrogen and oxygen mixture. The embryos were photographed in a culture coin dish made from polypropylene with a neutral media pH = 7. The inverted microscopy principle was used, with 20x lenses, without zoom, with focusing and with field of view of 350 um. A camera sensor used was IDS UI-3260CP-M-GL (IDS Imaging Development Systems GmbH, Obersulm, Germany). An example of embryo images in the Esco dataset is presented in Figure 4. The evaluation and training of GANs was done on an Intel i5-4570 CPU with a GeForce 1060 GPU and 8 GB of RAM.

### 3.2. Results

The training of GAN was carried out in 200,000 iterations, with a sample size of 256 images per batch. The duration of training using GeForce 1060 GPU was about 15 h 45 min.

**Figure 5:**
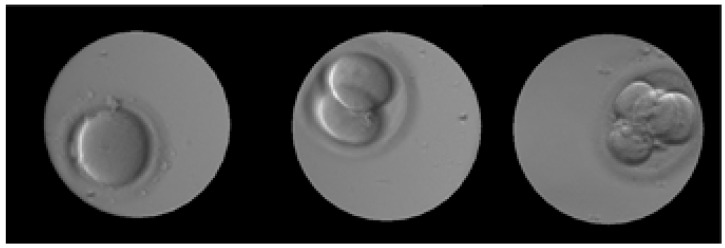
Example of final generated embryo cell images using the proposed algorithm. From left to right: One, two, and four cells. Images were filtered from “salt and pepper” noise using a median filter. See Table 1 for raw outputs.

From the final generated images, one can easily count the number of embryonic cells and determine the class (Figure 5). During subsequent iterations, the generator restored the embryo image and reduced the amount of noise in the generated images. 

During the training, embryo cell images were generated at every 25,000 iterations. An example of these images is shown in Table 1. 

After 25,000 iterations, we can see that he managed to restore the plate’s flair and release the background, but the embryo itself was not restored. After 100,000 iterations, the generator has been able to clearly reproduce the image of the embryo cell.

**Figure 6:**
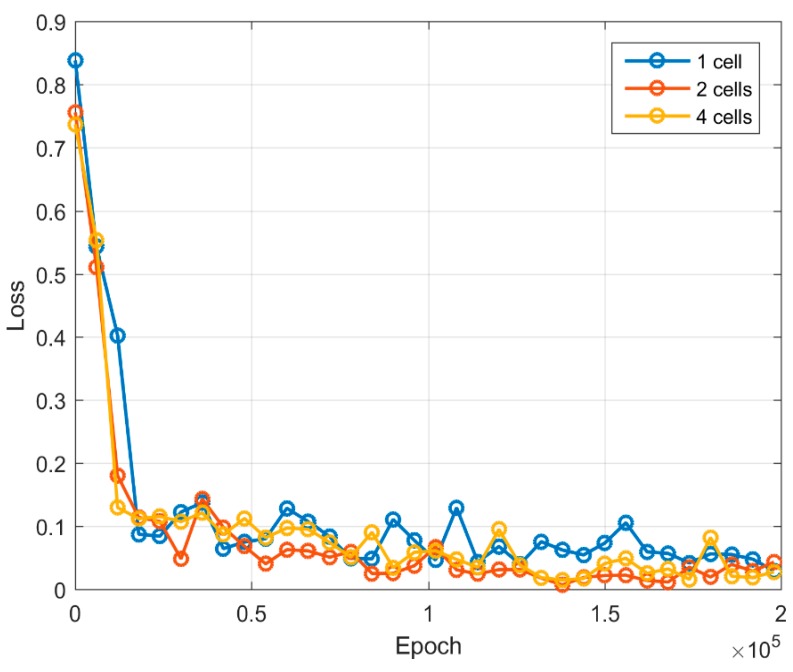
Loss log graph for the generator network.

**Figure 7:**
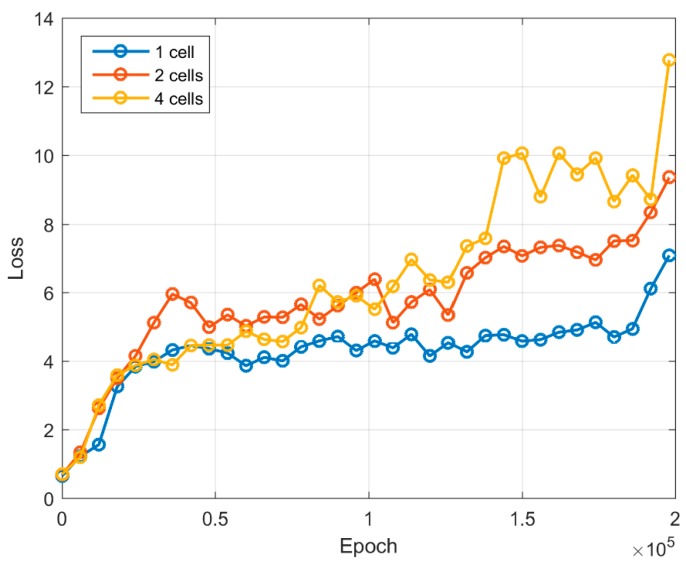
Loss log graph for the discriminator network.

The error function values for both generator and discriminator are shown in Figure 6 and Figure 7. During an initial training up to 20,000 iterations, the error function value of the generator increases rapidly. Further increase of the error function results in a slower pace. Once the training number reaches 75,000 iterations, the value of the generator error function begins to increase with the training of two-cell and four-cell images. The generator becomes able to produce more complex images, which is also visible from the generated sample images (see Table 1). The one-cell image can be identified from 50,000 iterations, whereas two-cell and four-cell images can be recognized from 75,000–125,000 iterations. From the discriminator error feature, we can see that starting from 20,000 iterations it becomes difficult to separate real images from artificially generated images, and the value of the error function is less than 0.15. A value of discriminator log loss increases, as discriminator is unable to differentiate between a real image and a generated image.

For the expert-based evaluation (see Table 2), 1500 images (500 in each class) were generated. The number of artificial one-cell images, where one cell was clearly recognized, was 96.2%. The quality of two-cell and four-cell image generation of more complex images deteriorated. Of the two-cell images, the number of cells could be clearly determined from 86.8% of the images. When evaluating the four-cell images, 80% accuracy was obtained, i.e., one out of five images was generated inaccurately (as decided by an expert).

**Figure 8:**
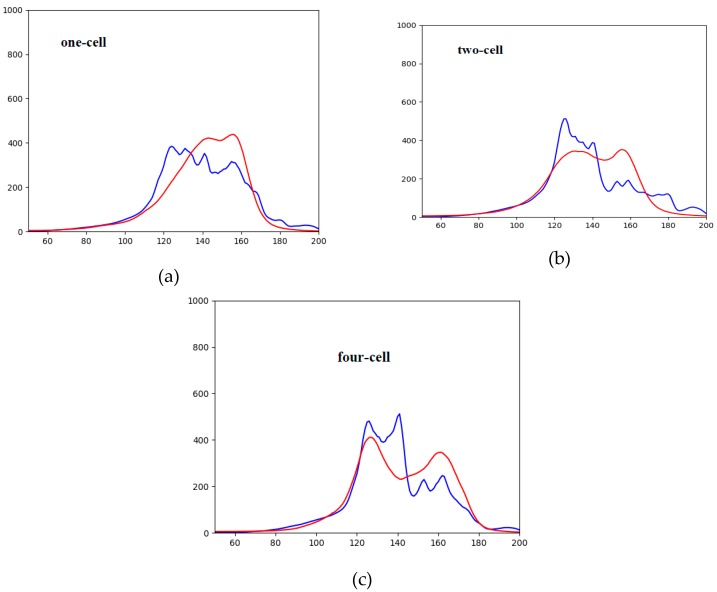
Comparison of histograms of real vs. generated images (real images—blue, generated images—red): (**a**) one-cell images, (**b**) two-cell images, (**c**) four-cell images.

Additionally, the image similarity was evaluated by comparing their histograms. In Figure 8, one can see the comparison of image histograms, where the blue curve is the average histogram of the training image dataset and the red is the average histogram of the artificially generated images. In all three classes, we can see that the generated image is brighter than the real one. This could be explained due to the slight “salt-and-pepper”-type noise in the generated image. The highest histogram match was obtained in the single-cell-generated images. This shows that the generator was better able to reproduce images of a simpler structure. Note that Figure 8 shows an evaluation of a generated cell image with a maximum epoch number of 200,000. The particular histogram cannot be identical as a single network generates images of different cells. This comparison was done by evaluating the average histogram of all (training and generated) image histograms.

Table 3 shows the results of a comparison of normalized histograms using correlation, Chi-square, intersection, and Bhattacharyya distance formulas. In all four cases, the largest histogram coincidence was shown by the one-cell generated images, while the two-cell and four-cell images performed worse.

We also compared the values of the textural (Haralick) features for the original and generated embryo images and evaluated the statistical difference between the values of features using the Student’s t-test. The results were significant (*p* < 0.01) for all Haralick features except the sum variance feature (one-cell images), variance and sum average (two-cell images), and the sum variance feature (four-cell images).

To compare the Haralick features from both (original and generated) image datasets, we also used the Pearson correlation between the principal components of feature sets extracted using principal component analysis (PCA). For both feature sets, the first principal component (PC1) explains more than 98% of variance, so we use only PC1 for further comparison. The results are presented in Table 4 and show a very high correlation between the PC vectors.

## 4. Discussion

As the correct comparison with other algorithms not possible due to very different source datasets used, we tried to compare within the misclassification rate, by comparing percentage of the images from all generated groups were assigned to correct class or not at all. Our HEMIGEN method was rated at 12.3%. 

Table 5 provides a comparison of the misclassification rate of synthetic images when compared to the results obtained by other authors. Gaussian mixture deep generative network (DGN) demonstrated 36.02% misclassification rate [51]. DGN with auxiliary variables and two stochastic layers and skip connections achieved the 16.61% misclassification rate [52]. Semi-supervised classification and image generation with four-layer generator demonstrated 8.11% misclassification rate on house number image generation [48]. The adversarial learned inference (ALI) model, which jointly learns a generation network and an inference network using an adversarial process, reached a misclassification rate of 7.42% using CIFAR10 test set (tiny images) [53]. The WGAN-based approaches ranged from 6.9% [21] to 50% [40] depending on the application. The methods indicated are only “loosely” comparable, taking into account the differences (importance) of features of synthetic image targeted and scopes of the works by other researchers.

We also checked for the problem of mode collapse where the generator would produce the same image over and over, thus fooling discriminator, while no new image would be generated in real-life. We have not found this case in our approach, possibly due to an adequate number of different embryos used. Some generated images had embryos, which overlap one another, but this was not considered a failure, but a realistic case of cell division.

## 5. Conclusions

We used generative adversarial networks trained on real human embryo cell images to generate a dataset of synthetic one-, two-, and four-cell embryo images. We have achieved the highest quality of generated images for single-cell embryo images, where 96.2% of the synthetic embryo images were recognized as accurate and usable by human experts. The worst accuracy was achieved for the synthetic four-cell images, of which only 80% could be identified correctly. These results were confirmed by the histogram comparison, which achieved the highest scores for synthetic single-cell images (an average correlation of 0.995 was achieved when comparing histograms of real and synthetic one-cell embryo images), as well as by comparison of image textures analyzed using the Haralick features. 

As our algorithm allows us to manipulate the size, position, and number of the artificially generated embryo cell images, these images can then be used to train and validate other embryo image processing algorithms, when the real embryo images are not available, or the number of available real embryo images is too small for training neural networks.

## Figures and Tables

**Table 1:** Evolution of embryo cell images during GAN training.

Epoch	0	25,000	50,000	75,000	100,000	125,000	150,000	175,000	200,000
one-cell	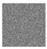	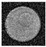	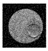	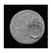	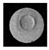	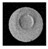	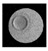	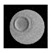	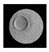
two-cells	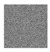	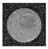	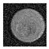	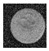	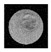	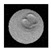	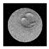	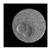	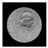
four-cells	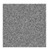	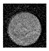	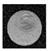	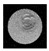	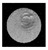	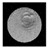	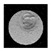	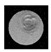	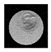

**Table 2:** Evaluation of the generated embryo image cells by human experts.

Class	Test Images (Generated)	Good Images	Accuracy by Expert Selection
one-cell	500	481	96.2%
two-cells	500	434	86.8%
four-cells	500	400	80.00%

**Table 3:** Evaluation of generated artificial embryo cell images using histogram comparison criteria.

	One-cell	Two-cells	Four-cells
Correlation	0.995	0.990	0.986
Chi-square	0.236	0.455	0.442
Intersection	1.883	1.849	1.873
Bhattacharyya	0.147	0.210	0.208

**Table 4:** Results of principal component analysis (PCA) of Haralick features from original and synthetic embryo images.

	One-cell	Two-cells	Four-cells
Explained variance of PC1 (original)	99.90%	99.94%	99.86%
Explained variance of PC1 (synthetic)	98.95%	99.60%	99.73%
Correlation between values of PC1 (original) and PC1 (synthetic)	1.0000	0.9997	0.9999

**Table 5:** Misclassification rate.

Model	Images Analyzed	Overall Misclassification Rate
DGN [51]	Digits 0 to 9 and combination (based on SVHN, and NORB sets) (*realistic images*)	36.02%
Skip Deep Generative Model [52]	Digits 0 to 9 and combination (based on SVHN, and NORB sets) (*realistic images*)	16.61%
GAN (feature matching) [48]	LSVRC2012 dataset with 1,000 categories (*non-realistic images*)	8.11%
ALI [53]	25 types of shapes (*non-realistic images*)	7.42%
WGAN [40]	6 classes of brain MR images (*realistic images*)	50%
WGAN-GP [21]	8 classes of florescent microscopy images (*realistic images*)	6.9%
HEMIGEN (our approach)	Once cell, Two cells, Four cells (*realistic images*)	12.3%
